# The impact of approaching grief on the neonatal team: professional experience report

**DOI:** 10.31744/einstein_journal/2022RC6698

**Published:** 2022-08-12

**Authors:** Cristiane Maria da Conceição Griffin, Jessica Macedo Pamponet, Viviane Bianca Bella, Gustavo Foronda, Soraya Gomiero Fonseca Azzi, Paula Alves Gonçalves

**Affiliations:** 1 Hospital Israelita Albert Einstein São Paulo SP Brazil Hospital Israelita Albert Einstein, São Paulo, SP, Brazil.

**Keywords:** Bereavement, Grief, Holistic nursing, Patient care team, Intensive care units, neonatal

## Abstract

This case report describes the holistic performance of the multidisciplinary team in a context of approaching the moment of death of a newborn, in a cross-cultural approach, and its impact on the working group. We report the clinical case of a neonate diagnosed as severe congenital heart disease, who evolved with early surgical intervention and died on the second day of life. Considering the neonatal intensive care unit an area requiring performance of a specialized team that addresses, with a high level of complexity, in a broad way, the patient, and his family, the death of a child breaks the logic of the life cycle. The multiprofessional and family management of the newborn in question illustrates the opportunities that are still being improved in the care of mourning in the neonatal period. Demonstrating that the care process does not end at the time of death, and the period of mourning is still a challenge for the neonatal team. Even so, parents can be welcomed, with care provided in a private and secure manner, with their needs, and preserved individuality. We observe the need for professional qualification and support for mourning and the need for specific institutional policies, since the health organizations must be attentive and take care of those who care, be cared for with a different look at their emotions.

## INTRODUCTION

The neonatal intensive care unit (NICU) requires specialized teams that address, with a high level of complexity and in a comprehensive manner, the patients and their families. The experience in care of critically ill patients that pediatricians and the multiprofessional team accumulate and develop over time has a direct impact on the experience that grieving parents will carry throughout life.^( 1 )^ The death of a child breaks the logic of the life cycle, and its sudden event may make it even more difficult to process the loss.^( 2 , 3 )^ Parents need to complete their bond with the neonate and gradually distance themselves from them, to focus on their own needs and family life, progressing in the grieving process.^( 4 )^

This report aims to describe a clinical case with holistic action of the multiprofessional neonatal team with a cross-cultural approach. It exemplifies an opportunity for improvement in the routine of the NICU related to care of health professionals with the direct caregivers and family, considering the importance of caring for those who care in an adverse situation, such as the death of the newborn.

This theme is very relevant, as it has a great impact on the practice of professionals and family members. There is a great need for the multiprofessional team to be trained, not only in the technical-scientific aspect, but also in non-violent communication and in the approach of difficult subjects, for the benefit of all who are present in the care. The literature states the death of the newborn for NICU professionals is an experience of conflicting and excruciating feelings, due to its depth.^( 5 )^ However, a scarcity of national articles on the subject is noted.

In view of these points, this paper proposes to contribute to the performance of the multiprofessional team at the time of death of a newborn. The methodology used for this study was the experience report, considered an important source of information to improve treatment of patients. The communication of this report is relevant because it deals with an unusual family approach, resulting in innovative care.

This report is of interest for the work of nurses in different areas, containing an analysis of conceptual implications and a description of procedures as intervention strategies.^( 6 )^ The setting of the present study was a tertiary level private hospital in São Paulo (SP), Brazil. The hospital had 617 beds and served several areas. The NICU had 22 beds and a multidisciplinary team consisting of nurses, nursing technicians, neonatologists, physical therapists, speech therapists, and psychologists, providing comprehensive care. The newborn was hospitalized at the NICU in 2019.

Data were collected indirectly and anonymously from the patient’s electronic medical record. A discussion of the case and its progression was held with the multiprofessional team, as well as the description of the report among the professionals who provided care to the patient. This project was approved by the Research Ethics Committee of *Hospital Israelita Albert Einstein* (HIAE), # 3.717.070, CAAE: 23460719.7.0000.0071. The criteria of Resolution 466, of 2012, of the National Health Council, which deals with research with human beings, were followed. The mandatory terms of presentation are in accordance with the resolutions in effect. The request for exemption of the informed consent forms was accepted, due to the absence of sensitive data that could identify the patient.

## CASE REPORT

A male newborn patient, third child. Intrauterine diagnosis of hypoplastic left heart syndrome, having undergone prenatal care with an obstetrician specialized in fetal medicine and congenital malformations and with a pediatric cardiologist. During the monthly prenatal consultations, the possibilities of surgical treatment, risks of complications, and statistical data regarding prognosis and death were discussed. The parents centralized the decisions although they had family support, and together with the prenatal team, they took into consideration the risks and benefits of each surgical technique. They opted for a hybrid procedure (stent implantation in the arterial canal topography and selective banding of the pulmonary arteries), but were against extracorporeal membrane oxygenation (ECMO), due to the associated risk of neurological sequelae.

He was born by vaginal delivery, at 38 6/7 weeks gestational age, weight 3,380g, Apgar score 8/9, and no intercurrent events. He was placed in skin-to-skin contact with his mother and had a late umbilical cord clamping. Accompanied by the father, he was examined, warmed up, maintaining oxygen saturation (SatO_2_) 85% at room air.

An immediate echocardiogram confirmed hypoplastic left heart syndrome with mitral stenosis and aortic atresia with restrictive atrial septal defect (ASD). A balloon catheter atrioseptostomy was indicated, with an increase of the ASD from 1.5mm to 5mm, and reduction of the trans-septal gradient from 29mmHg to 4mmHg.

In the NICU, he remained on invasive mechanical ventilation (IMV), analgesia, continuous prostaglandin infusion, and maintenance saline. Continuous adrenaline was introduced as a vasoactive drug after umbilical catheterization, with adequate volemia, and metabolic disorders were corrected. It was necessary to adjust the inspired oxygen fraction (FiO_2_) below the physiological value (21%) to improve the systemic and pulmonary flow (Qp/Qs) ratio. To this end, nitrogen was added to the mixture of ventilatory gases, obtaining FiO_2_ of 19%, with improvement of coronary perfusion and systemic output.

The parents had free access to the newborn, the mother was encouraged to carry the baby, according to the NICU routine. Knowing about the early surgical schedule, the multiprofessional team also focused on parental care. Constant contact between parents and the newborn was promoted, psychological parental support was provided, and continuous dialogue was maintained regarding doubts and feelings experienced by the parents. The mother was encouraged to attend the milk bank, so that breastfeeding would be preponderant as soon as possible, always aware of the seriousness of the situation and of the newborn’s prognosis. All along, the decisions about type of treatment were centered on the parents, with all risks being clarified by the attending physician. After parental consent and clarification of all doubts, the parents’ decision was respected considering their autonomy aiming at ethics and patient safety.

A hybrid procedure was performed on the second day of life, which included selective banding of the pulmonary arteries via sternotomy and stenting of the arterial canal in the cath lab; invasive blood pressure monitoring; placement of a central venous catheter; and allocation of a mediastinal drain, and pacemaker wires.

He returned to the NICU on adrenaline and IMV, requiring volume and metabolic adjustment and transfusion of blood products. Two hours later, he presented sudden instability with cardiac arrest.

Neonatal resuscitation was initiated, with chest compressions, manual ventilation, use of standard doses of vasoactive drugs, and volume expansion. The echocardiogram showed severe dysfunction, no pericardial effusion, and radiography ruled out air leak syndrome. The probable diagnosis considered was acute myocardial infarction, considering the anatomy of the congenital heart disease and physiology after the surgical procedure. Starting on ECMO was considered, but the previous parental resolution regarding the non-use of this method of hemodynamic support was respected.

The mother was summoned to the NICU and arrived during the resuscitation process, which lasted approximately 40 minutes with no response, and death was confirmed. Upon being informed about the clinical progression, with prolonged resuscitation and no effective response, the mother immediately agreed to interrupting the procedures and requested to remove the newborn from the incubator. The mother physically distanced herself from the healthcare professionals, remained with her son on her lap, and tried to remove all invasive devices from her son (catheters, drains, accesses, and probes).

Slowly, the nursing staff explained to the mother, in her native language, the need and importance of assisting her in this process. Respecting the organizational protocols, the trained professionals removed the patient’s devices, following the good practices and safety standards for the patient, the family member, and the health professionals. Thus, the nursing and medical staff, with the active participation of the mother, removed the devices, one by one. The mother was welcomed, held the newborn in her arms, and received the comfort of the team’s words in her language of understanding. On her lap, the newborn remained for about 90 minutes, when the mother then requested the opportunity to change the newborn’s diaper. Faced with such a simple request, which resulted in the diaper changed by the mother, the multiprofessional team could see the great significance of such apparently small attitudes. There were several benefits brought by this practice, among them answering a last request from a grieving mother, humanizing the moment of death, and bringing a new look to issues that are so difficult to be addressed - since this practice was innovative in the NICU.

Because family and religion are two entities that can help in the grieving process, the parents were offered the possibility of the team contacting other family members, but the parents chose to conduct the communication with the grandparents. Regarding the possibility of counseling or religious ceremony, the parents refused such support.^( 7 )^

Following the protocol of the event, the newborn would be transferred to the morgue, where it would remain until transportation for the wake and burial. However, knowing that culturally, in her country of origin, the mother would take her newborn to be mourned at home and only then be sent to the cemetery, the team understood the mother’s request to keep her son wrapped in her scarf and in her arms until the removal to the wake, allowing the newborn and mother to be safe and accompanied until the moment of transfer - which is hospital standard - and enabling the mother’s aspiration to remain with her son as long as desired.

## DISCUSSION

Changing a diaper, followed by the warmth of a lap, is care that consolidates the love between mother and newborn. Allowing parents to express their wishes regarding the newborn’s care plan polishes and keeps the assistance proposed to the newborn in constant development. Therefore, it is necessary to adjust organizational routines and protocols that safely and responsibly include family participation, considering both the newborn’s diagnosis and the religious, cultural, social, and psychological idiosyncrasies in which they are inserted.

The literature reports nurses prepared to deal with grief provide better care; however, organizations that offer support for neonatal nurses experiencing grief and loss are not a standard.^( 8 )^ With a new pattern of thought, the John Hopkins Children’s Center offers grief reporting sessions to support bereaved pediatric nurses to understand the patient’s loss.^( 9 )^ With the purpose of providing freedom of expression about feelings, coping strategies are significant to provide better grief management.^( 9 )^

Patients who died suddenly or who have been under the care of the staff for a long period are described as the most difficult losses. Learning to cope with grief and patient loss is an essential skill, but one that is poorly emphasized and developed among nurses.

The multiprofessional team was open to adaptations for the humanized care of the grieving family and transformed this experience into learning, hoping to promote the best experience for the neonatal bereaved patient and his family. This represents an advance in the practice of facing neonatal death. There was movement of the entire unit group to assist the on-duty nurse, both in the administrative part and during the process of cardiopulmonary resuscitation (CPR) and family support. A fundamental point of support, humanization, and teamwork was observed, demonstrating the difference due to the impact of the situation experienced by the team.

The loss of a child is one of the most overwhelming events for human beings. Death in the early neonatal period is the interruption of dreams and expectations built during pregnancy. For the bereavement process to occur, it is necessary to face the facts and recognize the importance of performing all the rituals.^( 10 , 11 )^ The welcoming and empathy towards the demands do not neutralize the pain, but contribute to make the suffering bearable, and the mourning process occurs gradually.

It is important to listen actively and to consider the individualities in the face of loss, respecting the family unit, without a judgmental posture or externalization of prejudice.^( 12 )^ Nursing is essential in this process, for it knows and values the family needs, understands the suffering related to the loss, and offers support and respect in the moment of pain, establishing a link of trust with the family.^( 13 )^

The multiprofessional team demonstrated synergy and empathy in the compassionate action, fulfilling the mother’s wishes, with a determining role in the parents’ experience. The team, even around the fragility of the events and with the expertise acquired over years of experience, respected and added to the mother’s right to intervene in the newborn’s care. The validation of the mother’s understanding that the parents are the most important caregivers for the child allowed, even during this moment of intense emotion, the creation of lasting memories. Therefore, even with all their accumulated knowledge, the health team supported the parental exercise of neonatal care, reassuring and calming the parents until the end of their child’s life.^( 14 )^The report is an example in line with the literature, which suggests the continuous development of skills to care for the grief of parents who have lost their newborn, creating a reconnection between the parties and alleviating the pain through empathy and family-centered care,^( 15 )^ as well as highlighting the importance of empathy towards the feelings experienced by the team, and the provision of adequate emotional support.

**Figure 1:**
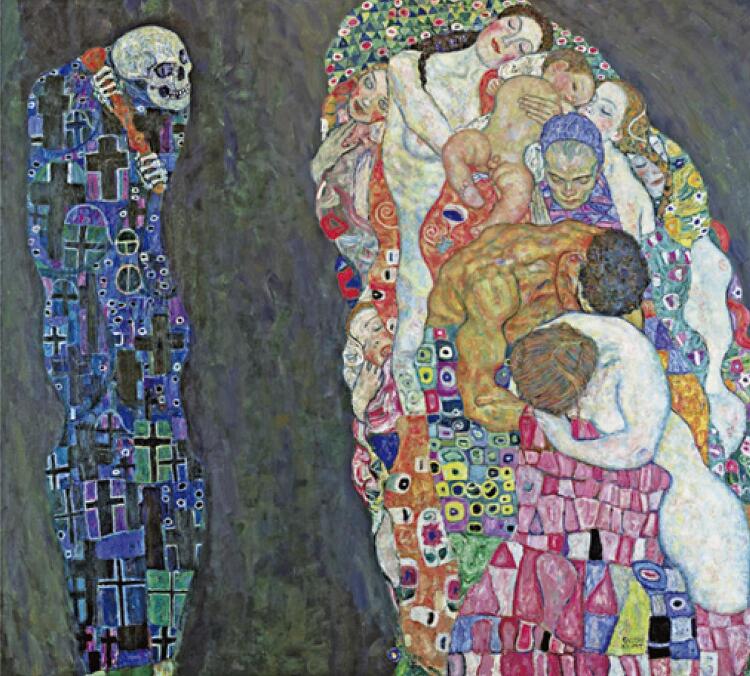
Death and life, by Gustav Klimt, 1910 Available from: https://www.leopoldmuseum.org/en/collection/highlights/146^(16)^

Source: Klimt G. Death and life. Wien (AU): Leopold Museum; 1911 [cited 2021 May 5].

Gustav Klimt’s magnificent work Death and Life ( Figure 1 ),^( 16 )^ alludes to the modern dance of death, inducing a note of hope and reconciliation, for instead of feeling threatened by the figure of death, human beings seem to welcome it. The artist’s imagination no longer focuses on the physical union, but on the expectation that precedes it.^( 17 )^This representation of the proximity of death seems to convey the intensity of emotions and feelings of the team and the family when caring for the farewell of a newborn, already known to have little chance of survival since the prenatal period. This leads us to question what care is offered to the team that takes care of the death process. How are these professionals prepared to take care of their own pain? Are we aware of the emotional issues of the professionals who are faced with the care of death?.

## CONCLUSION

This experience as a team demonstrates that the moment of bereavement is a challenge for the neonatal team. In addition to welcoming, parents need access to privacy, with their needs and individuality being respected in a holistic and humanized manner during neonatal bereavement. This performance was of great positive impact, even in such a difficult moment, with the ability to adjust routines and protocols, allowing the active participation of the neonate’s family, which is essential for processing grief by parents who lose their newborn. There was an opportunity to receive feedback from the parents, who thanked us for all the support and care given to the newborn and the family during the process, respecting them even during suffering. It was shown that the process of caring does not end at the time of death, but goes beyond and can have a positive impact on the families. In addition to all these points of contribution, the multiprofessional team needs to be seen in this process with a new look of empathy, indicating the need for specific professional qualification regarding grief support, and the need for organizational policies focused on these care processes, in addition to the contribution of new reports and studies on the subject.
